# Iodine status of pregnant women in Northern Cyprus

**DOI:** 10.4274/balkanmedj.2018.0679

**Published:** 2018-11-15

**Authors:** Umut Mousa, Hasan Sav, Osman Köseoğluları, Murat Faik Erdoğan

**Affiliations:** 1 Department of Endocrinology and Metabolism, Dr. Burhan Nalbantoğlu State Hospital, Nicosia, Cyprus; 2 Marmara Clinic, Lefkoşa, Cyprus; 3 Department of Endocrinology and Metabolism, Ankara University School of Medicine, Ankara, Turkey

To the Editor,

Iodine requirements increase in pregnancy (1). The World Health Organization and U.S. Institute of Medicine suggest a daily intake of 250 µg/d (2) and 220 µg/d, respectively, for pregnant women (3). A median urinary iodine concentration below 150 µg/L reflects insufficient iodine intake in a pregnant population (2).

Before the introduction of iodized salt to our country in 1999, we performed a study in 625 school-age children and found that the median (minimum-maximum) urinary iodine concentration was 120 (11-900) µg/L (4). In 2003, we conducted a monitoring study in 450 school-age children. The median (minimum-maximum) urinary iodine concentration increased to 139.5 (16-770) µg/L (unpublished data). Thus, our region was considered to be iodine-replete. We have no data regarding pregnant subjects. The aim of this study was to analyze the urinary iodine concentration in a cohort of pregnant women residing in Northern Cyprus.

The study was approved by “B. Nalbantoglu Ethical Committee, Nicosia, Cyprus” in which 258 pregnant inhabitants in all trimesters were included between January and June 2016.

We administered a questionnaire and obtained morning iodine samples from the study population until laboratory analysis performed in Ankara University, Medical School, Endocrinology Laboratory, which is a part of the Ensuring the Quality of Urinary Iodine Procedures program conducted by the Centers for Disease Control and Prevention (USA).

Subjects using iodine supplements or iodine-containing multivitamins were not eligible for this study.

According to the World Health Organization criteria for iodine nutrition during pregnancy, participants were divided into five groups: adequate iodine (urinary iodine concentration: 150-249 μg/L), mild deficiency (urinary iodine concentration: 100-150 μg/L), moderate deficiency (urinary iodine concentration <100 μg/L), severe deficiency (urinary iodine concentration <50 μg/L), and more than adequate or excessive (urinary iodine concentration ≥250 μg/L) groups. General characteristics of the study group are presented in Table 1. The mean age of the study group was 28.62±5.8 years (17-46). The median (minimum-maximum) urinary iodine concentration was 110 (8-450) µg/L. The median urinary iodine concentration were similar in all three trimesters (1st trimester 108 µg/L, 2nd trimester 112 µg/L, 3rd trimester 116 µg/L; p=0.392).

The median urinary iodine concentration was 113 µg/L among subjects using iodized salt and 77 µg/L in subjects using noniodized salt (p=0.330). The median urinary iodine concentration was 114 µg/L among subjects who restricted their salt intake and 106 µg/L among those who did not restrict their salt intake (p=0.273). The median urinary iodine concentration was 102 µg/L for those with a history of spontaneous abortion and 111 µg/L for those with no abortion history (p=0.293).

This study is the first to report on the iodine status of pregnant women in Northern Cyprus. The urinary iodine concentration value in this population is insufficient for pregnant women according to World Health Organization guidelines. Many studies have reported insufficient urinary iodine concentration in pregnancy, even in areas of iodine sufficiency (5).

Routine iodine supplementation during pregnancy is recommended by many authorities worldwide, even in iodine-sufficient areas (3). Significantly low maternal iodine intake during pregnancy can lead to irreversible neurological damage and cretinism (1,2,3,4,5). Maternal iodine insufficiency has been previously linked to abortion and preterm birth (1,2,3,4,5). However, in this study the median urinary iodine concentration was similar in subjects with and without a previous history of abortion.

We conclude that, although Northern Cyprus was proven to be an iodine-replete population, iodine nutrition is insufficient among pregnant women, and because more than 50% of our subjects have urinary iodine concentration <100 μg/L, we need to advocate for the use routine supplementation of iodine at 100-150 µg/d together with iodized salt usage.

## Figures and Tables

**Table 1:**
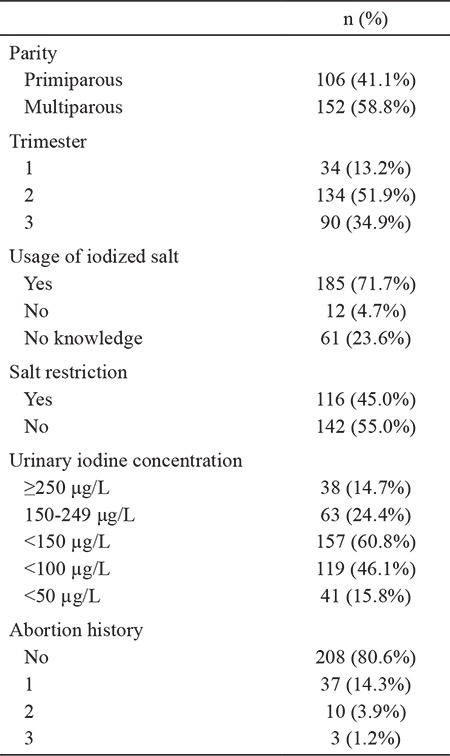
General characteristics of the study population
